# Posterior Inferior Cerebellar Infarct in a Younger Adult Male with Vertigo and Ataxia

**DOI:** 10.51894/001c.6385

**Published:** 2017-12-19

**Authors:** Angela J. VanWagner, Benjamin Doerr, Stephanie Hernandez

**Affiliations:** 1 McLaren Macomb, PGY2 Emergency Medicine Resident; 2 McLaren Macomb, Emergency Medicine Attending Physician

**Keywords:** neurological deficit, ataxia, vertigo, cerebellar infarct

## Abstract

Vertigo is a common complaint in patients who present to the emergency department. It can be a manifestation originating from several different disease processes. Although most patients with vertigo, especially younger patients, will have a benign disorder, up to 3% of such patients will have a cerebellar infarct. Although ruling out these types of fatal diagnoses is essential for emergency medicine physicians, this task can be especially complicated. Classic signs of a cerebellar infarct include symptoms suggestive of central vertigo with focal neurologic deficits on physical exam. Up to 10% of patients with cerebellar infarctions, however, present to the emergency department with vertigo and no focal neurologic deficits. The following case report discusses a male in his late twenties with the chief complaint of vertigo. On initial exam, he had no focal neurologic deficits but did have other concerning symptoms including severe ataxia. Imaging subsequently revealed the patient to have sustained a cerebellar infarct. When differentiating benign forms of vertigo from cerebellar infarcts or other central causes, the clinician should take into account risk factors such as central symptoms including neurologic deficits and severe ataxia. Implementing this strategy may decrease morbidity and mortality associated with cerebellar infarctions.

## INTRODUCTION

Vertigo is a common complaint in patients presenting to the emergency department, as well as other outpatient settings. Vertigo has been defined as a pathologic illusion of movement.^1^ A first step in evaluating a patient with vertigo is distinguishing between central and peripheral etiologies.^2^ Clinicians can evaluate symptom onset, intensity, duration, direction of nystagmus (i.e., involuntary eye movement), associated neurologic findings and auditory findings as well as positional effect.^2^ Peripheral vertigo is generally characterized by sudden onset and severe intensity which last seconds to minutes. In this type of vertigo, nystagmus is classically horizontal with symptoms exacerbated by head position. Peripheral vertigo generally lacks neurologic findings, although there may be tinnitus (i.e., perception of ringing in ears) or decreased hearing. Peripheral etiologies include benign paroxysmal positional vertigo, labyrinthitis, Meniere’s disease, vestibular neuritis, and acoustic neuroma.^3^

Central vertigo can present gradually and mild in intensity and continue for weeks to months. It may also present as sudden and severe in nature, lasting seconds to minutes. Other characteristics of central vertigo include nystagmus in any direction, symptoms not exacerbated by body position, and the presence of neurologic findings. Central causes are generally more serious and include vascular disorders such as cerebellar masses, hemorrhage and infarction.^3^ It is therefore crucial to differentiate between central and peripheral vertigo as certain causes of central vertigo can be fatal when left untreated.

The posterior inferior cerebellar artery (PICA) is a branch of the vertebral artery and one of three supplying arteries to the cerebellum. The other two branches are the anterior inferior cerebellar artery (AICA) as well as the superior cerebellar artery (SCA). A large infarction of the PICA classically causes symptoms of lateral medullary syndrome (i.e., Wallenberg syndrome). These symptoms include vertigo, ipsilateral facial numbness, loss of corneal reflex, Horner’s syndrome (i.e., ipsilateral miosis, ptosis and anhidrosis), pharyngeal and laryngeal paralysis, and contralateral loss of pain and temperature sensation in the extremities.^1^

Not all patients, however, present with these symptoms. Up to 10% of patients with cerebellar infarctions will present with vertigo and no other focal neurologic deficits.^3^ This is significant as the clinical consequences of cerebellar infarctions can be serious and demand prompt medical attention. These complications include obstructive hydrocephalus, brainstem compression and subsequent death. Macdonell et al. analyzed cerebellar infarctions in patients to determine the frequency of life-threatening events and found that the fatality rate of cerebellar infarction was greater than other forms of brain infarction.^4^ These risks can be decreased by early recognition, strict monitoring and neurosurgical intervention if complications develop.

The diagnostic imaging tests of choice are currently Magnetic Resonance Imaging (MRI) and Magnetic Resonance Angiography (MRA) images of the brain.^5^ Computed Tomography (CT) scan combined with Computed Tomography Angiography (CTA) of the head are generally the initial imaging modalities used, although an infarction cannot be excluded as the sensitivity of CT has been shown to be only 26% in acute ischemic stroke.^3,6^ This makes a thorough patient history collection and physical examination critical during their diagnosis for vertigo.

Although providers should conduct a further workup on emergency department patients with possible central vertigo symptoms, patients may report frequently overlapping symptomatic findings.^1^ The most common cause of cerebellar infarction in all age groups is cardio embolic events and atherosclerosis.^5^ Therefore, risk factors are also used to select patients predisposed to central etiologies as a cause of their vertigo. These include hypertension, diabetes mellitus, smoking and a history of vascular disease.^5^

A study by Furman et al. in 2015 recommended immediate neuroimaging be obtained in older patients presenting with acute sustained vertigo and vascular risk factors, new severe headaches, or whose examinations are not typical for peripheral vestibulopathy.^5^ In patients younger than 40, the major etiology of cerebellar infarct is vascular events (67%), most commonly intracranial vertebral artery dissection, followed by cardioembolic events (20%), (e.g., a paradoxical emboli from a patent foramen ovale (PFO)).^7^ Additional risk factors include hypercoagulable disease states (e.g., Factor V Leiden, malignancy, Protein C and S deficiency) as well as recent head or neck trauma.

### Case Report

An African American male in his later 20s presented to the McLaren Macomb Emergency Department with his mother complaining of vertigo and nausea and vomiting for at least four hours. These symptoms had begun at approximately 4:00 am before his arrival. At that time, he awoke and felt the sensation that the room was spinning around him. He immediately noticed a headache located mainly in the left frontal-temporal region. He attempted to get out of bed and had difficulty ambulating secondary to severe vertigo. At this point, he had multiple episodes of vomiting. He first presented to the emergency department at approximately 8:00 am. The authors evaluated and treated him with anti-nausea medication, as well as intravenous fluid hydration and Ativan. He was determined to be stable for discharge with documented resolution of symptoms and sent home with a prescription for Antivert for symptoms as needed.

Once home, he reported significant worsening of his symptoms. The patient was having an increasingly difficult time ambulating, with worsening intractable nausea and vomiting as well as increasing headache. His mother then brought him back to the emergency department for further evaluation at about 11:00 pm. He denied any neck pain, head or neck trauma, numbness or weakness in his extremities, visual changes, chest pain or dyspnea. He denied ever having symptoms like this in the recent past.

Past medical history revealed the patient had hypertension but was not currently on any medical treatment. He denied any surgeries in the past. He did admit to active tobacco use (i.e., a pack per day for seven years) but denied illicit drug use or alcohol use. His maternal grandmother had sustained at least one transient ischemic attack, and his mother suffered from occasional migraines.

On presentation, the patient’s vital signs were as follows: blood pressure 138/80, temperature 97.7, pulse 86, respirations 16, and oxygen saturation 98%. Physical exam revealed the patient to appear uncomfortable, grimacing in pain due to his headache and laying in the left lateral decubitus position as he stated this helped his symptoms. Neurologic examination was unremarkable except for noted ataxia. He had no limb ataxia, and no evidence of cranial nerve, motor or sensation deficits. The patient did attempt to walk but needed support in order to ambulate in the emergency department. The authors found no meningeal signs. There was no appreciable nystagmus, and the Dix-Hallpike maneuver (a physical exam test used to identify benign paroxysmal positional vertigo) was negative.

**Figure 1:**
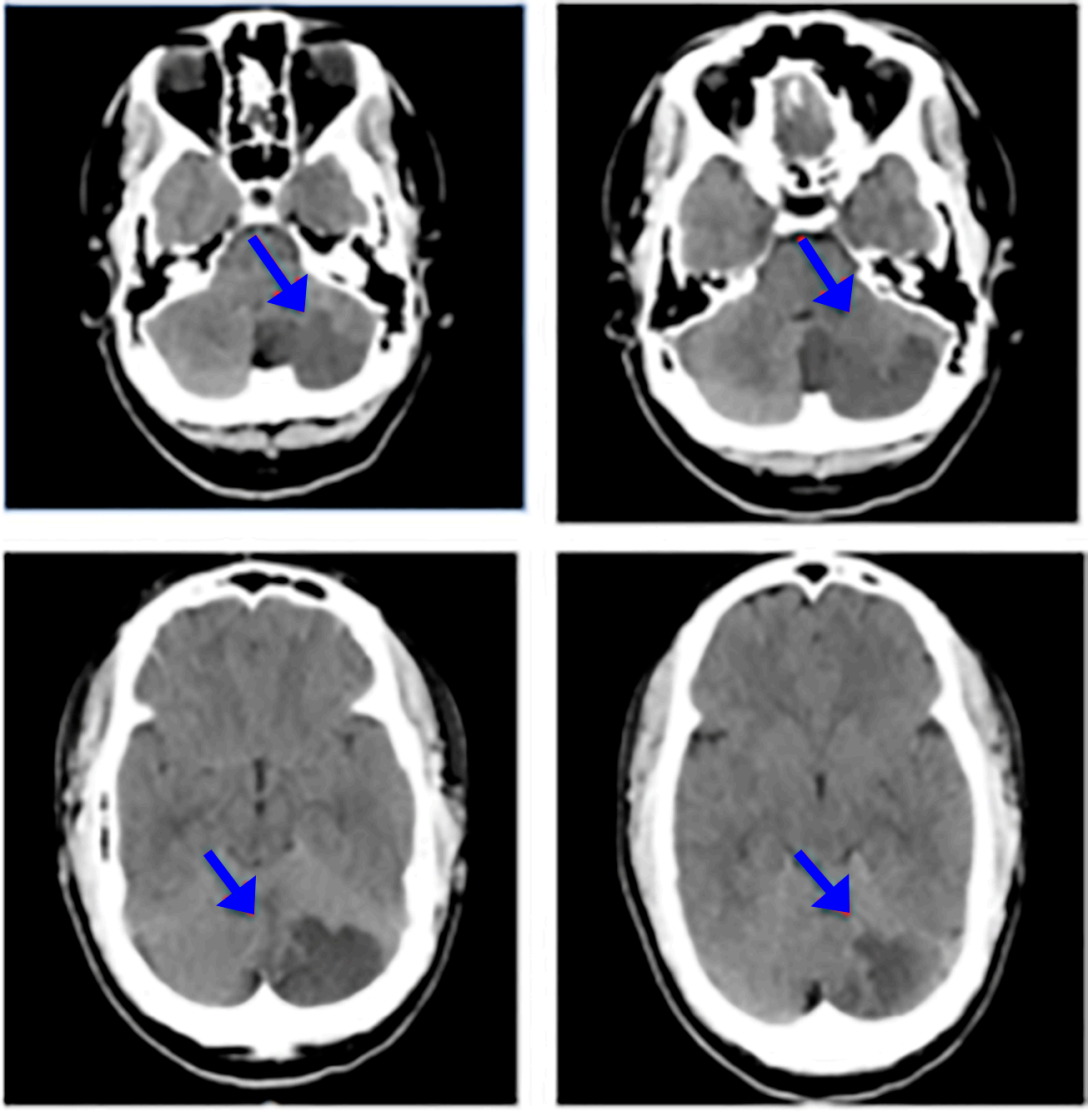
CT Demonstrating an Acute Left Cerebellar Infarct in the Distribution of the Posterior Inferior Cerebellar Artery. Pictures are from Caudal (top left) to Cranial (Bottom Right) Direction.

Laboratory studies showed a somewhat elevated white blood cell count of 13.7mcL. All other laboratory values were within normal limits. The authors ordered a CT and CTA of the brain that showed an area of hypo-attenuation with loss of the gray-white matter junction in the left cerebellum. This was consistent with an ischemic infarction along the distribution of the posterior inferior cerebellar artery. (Figure 1)

The patient was administered 325 mg aspirin and a MRI and MRA of the brain was then ordered. The patient was no longer a candidate for tissue plasminogen activator (TPA), (i.e., a thrombolytic agent in ischemic strokes) since he was beyond the therapeutic treatment window. The authors admitted him to the hospital Intensive Care Unit with frequent neurology checks as well as consults to interventional neurology, neurology and neurosurgery.

**Figure 2:**
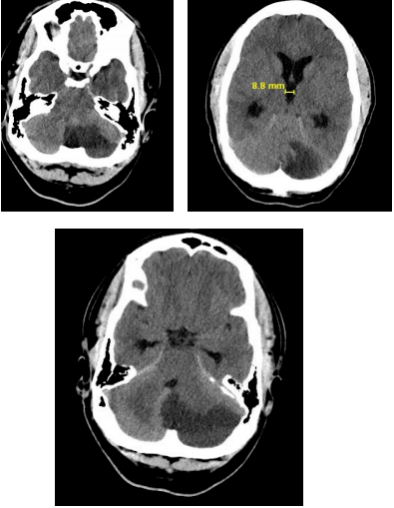
CT Demonstrating Progressed Dilation of Third and Lateral Ventricles.

They also obtained further imagining including CTA of the neck, results of which were unremarkable. The MRI and MRA of the brain showed a large subacute left cerebellar infarct with mass effect and narrowing of the fourth ventricle without any evidence of hydrocephalus. A repeat CT brain was also ordered the next day, demonstrating increased dilation of third and lateral ventricles. (Figure 2)

On the first day after hospital admission, the patient became more somnolent (i.e., sleepy, drowsy) and was diagnosed with obstructive hydrocephalous by neurosurgery. He went to the operating room for a right frontal external ventricular drain and his symptoms slowly improved throughout the rest of his stay. The hematology service also evaluated the patient for a possible hypercoagulable state. This workup was negative except for elevated antithrombin-III level as well as heterozygous MTHFR gene, which was not likely the cause of the infarct per hematology. The authors next obtained a transthoracic echocardiogram with bubble study. This test involves injection of agitated saline into a vein followed by a heart echocardiogram, allowing the physician to visualize direction of blood through the heart chambers. The results of this study were suggestive of a patent foramen ovale possibly putting the patient at risk for a paradoxical emboli.

After hospital Day 14, the patient was discharged with recommendations for tobacco cessation and was started on an antihypertensive medication. He was instructed to attend follow up clinic appointments at suggested intervals. At the time of discharge, the patient was no longer ataxic, and was without any neurologic deficits and deemed stable by all consultants.

## DISCUSSION

This case demonstrates the difficulty in distinguishing between cerebellar infarctions and other benign forms of vertigo in the emergency department and in outpatient medical settings. Clinicians use central vertigo symptoms as well as risk stratification to determine those in need of further workup, although cerebellar infarcts have the ability to present with isolated vertigo in patients without significant risk factors. For this reason, studies have shown the misdiagnosis of cerebellar infarcts in emergency departments to be about 35%.^3^

On initial physical exam, this particular patient had no focal neurologic abnormalities. Furthermore, classifying this patient correctly into central vertigo had been difficult since he possessed many characteristics of peripheral vertigo. (Table 1). His symptoms were sudden in onset and severe in intensity and had persisted for hours. His vertigo was somewhat positional as laying on his left side seemed to improve his symptoms. He revealed no focal neurologic deficits on exam, and there was no obvious nystagmus appreciated. With the exception of his hypertension and tobacco use, this patient was an otherwise healthy male in his late twenties.

**Table 1:** Characteristics of Peripheral and Central Ataxia

**Characteristic**	**Peripheral**	**Central**
**Onset**	Sudden	Gradual or sudden
**Intensity**	Severe	Mild
**Duration of Symptoms**	Usually seconds to minutes; Intermittent in nature	Usually weeks, months; Continuous in nature
**Direction of nystagmus**	One direction, usually horizontal	Vertical, down beating
**Effect of head position**	Worsened by position, often single critical position	Little change, associated with more than one position
**Neurologic findings**	None	Usually present
**Auditory findings**	May be present, including tinnitus	None

Although this patient did possess these two risk factors, his young age may have deterred clinicians from considering a more serious diagnosis such as cerebellar infarction. He had not sustained any recent neck or head trauma nor had a known history of hypercoagulable disease. Although providers had found him to have a patent foramen ovale during his hospital stay, this information was not available in the initial workup and management in the emergency department. Risk stratification in this instance can help little in helping providers decide whether neuroimaging would be beneficial. This patient could have been discharged home without further imaging based on his lack of central symptoms as well as relative young age and lower risk factors.

A 2015 review by Nelson et al highlights the importance of other physical findings that should be considered during clinical decision making for the vertiginous patient with severe ataxia and direction changing nystagmus.^8^ Although many patients with vertigo will present with some form of ataxia, general vertigo of central origin impairs their gait to a greater degree than peripheral causes.^5^ Patients with peripheral vertigo tend to be hesitant to move as this increases their symptoms, but are usually able to ambulate without assistance. Conversely, 71% of patients with cerebellar infarctions and isolated vertigo will also present with inability to ambulate without support.^9^

This particular patient had acute, severe ataxia that did not resolve during his emergency department stay. The acute onset of his ataxia should have also raised concern, as ataxia of sudden onset suggests cerebellar hemorrhage or infarction, while ataxia that is slowly progressive suggests chronic cerebellar disorders.^4^ This highlights the need for imaging in patients first presenting with severe ataxia and a chief complaint of vertigo, even in the absence of significant risk factors and other central symptoms. Although this patient did not present with any focal neurologic deficits and his vertigo seemed to have many characteristics of peripheral vertigo, he was unable to ambulate without assistance in the emergency department warranting further neurologic imaging including MRI and MRA.

## CONCLUSIONS

This report highlights a case of a male in his late twenties who presented to the emergency department with vertigo and severe ataxia. Despite the lack of focal neurologic deficits upon presentation and relative low risk of the patient, his providers subsequently obtained a CTA of the brain that showed a left cerebellar infarct in the distribution of the PICA. It can be very difficult to differentiate benign forms of vertigo from life threatening forms that require immediate medical attention.

Providers should attempt to classify patients based on their peripheral versus central symptoms. This can be complicated since patients may have overlapping symptoms. Providers should consider additional risk factors, with patients possessing several key risk factors warranting neuroimaging. This case demonstrates that despite diligence with the above strategies, some patients with cerebellar infarctions may be misdiagnosed. Physicians should focus on other patient symptoms, such as acute, severe ataxia, when distinguishing peripheral vertigo from cerebellar infarcts or other central causes. One can argue that providers should always order further diagnostic imaging including MRI and MRA of the brain for patients with vertigo and acute, severe ataxia.

### Conflict of Interest

The authors declare no conflict of interest.
